# Impact of the practice of touch-massage® by a nurse on the anxiety of patients with hematological disorders hospitalized in a sterile environment, a randomized, controlled study

**DOI:** 10.1186/s12906-023-04302-3

**Published:** 2024-01-02

**Authors:** Armelle Simon, Jean-Julien Nizard, Patrice Chevalier, Steven Le Gouill, Thomas Rulleau, Lucie Planche, Adrien Evin

**Affiliations:** 1 Nantes Université, CHU Nantes, Service Interdisciplinaire Douleur, Soins Palliatifs et de Support, Médecine intégrative, UIC 22, Nantes, F-44000 France; 2 Université Paris Est, EA4391 Therapeutic and Nervous Excitability, Creteil, F-93000 France; 3 Nantes Université, CHU Nantes, Hematology Department, Nantes, F-44000 France; 4 Nantes Université, CHU Nantes, CRCI2NA - INSERM UMR1307, CNRS UMR 6075, Equipe 12, Nantes, F-44000 France; 5 Institut Curie, Paris, France; 6 Université Paris Versailles Saint-Quentin, Versailles, France; 7 Nantes Université, CHU Nantes, Direction de la Recherche et l’Innovation, Coordination Générale des Soins, Nantes, F-44000 France; 8 Methodology and Biostatistics Unit, DRCI CHU Nantes CHD Vendée, La Roche Sur Yon, F-85000 France

**Keywords:** Massage, Anxiety, Hematology, Cancerology, Supportive care

## Abstract

**Context:**

In addition to curative care, supportive care is beneficial in managing the anxiety symptoms common in patients in sterile hematology unit. We hypothesize that personal massage can help the patient, particularly in this isolated setting where physical contact is extremely limited. The main objective of this study was to show that anxiety could be reduced after a touch-massage® performed by a nurse trained in this therapy.

**Methods:**

A single-center, randomized, unblinded controlled study in the sterile hematology unit of a French university hospital, validated by an ethics committee. The patients, aged between 18 and 65 years old, and suffering from a serious and progressive hematological pathology, were hospitalized in sterile hematology unit for a minimum of three weeks, patients were randomized into either a group receiving 15-minute touch-massage® sessions or a control group receiving an equivalent amount of quiet time once a week for three weeks. In the treated group, anxiety was assessed before and after each touch-massage® session, using the State-Trait Anxiety Inventory questionnaire with subscale state (STAI-State). In the control group, anxiety was assessed before and after a 15-minute quiet period. For each patient, the difference in the STAI-State score before and after each session (or period) was calculated, the primary endpoint was based on the average of these three differences. Each patient completed the Rosenberg Self-Esteem Questionnaire before the first session and after the last session.

**Results:**

Sixty-two patients were randomized. Touch-massage® significantly decreased patient anxiety: a mean decrease in STAI-State scale score of 10.6 [7.65–13.54] was obtained for the massage group (p ≤ 0.001) compared with the control group. The improvement in self-esteem score was not significant.

**Conclusion:**

This study provides convincing evidence for integrating touch-massage® in the treatment of patients in sterile hematology unit.

**Trial registration:**

NCT02343965.

**Supplementary Information:**

The online version contains supplementary material available at 10.1186/s12906-023-04302-3.

## Background

Many studies have been carried out on the considerable anxiety [1] frequently observed in patients undergoing cancer treatment, as well as the impact of treatment on the quality of life [2] of patients or on the subsequent development of depression [1] and Post-Traumatic Stress Symptoms [3]. The alteration of self-esteem, particularly during chemotherapy, leading to numerous physical changes, has also been reported [4] and need to be considered [5].

The recent diagnosis of a serious and progressive hematological disease leads the patient to rapid hospital care, due to the urgency of the treatment. In a few hours or days, the patient may go from being a “healthy individual” to being a “seriously ill patient”. Because of the strict hygiene rules, hospitalization in protective isolation means that the patient must discard all personal effects for a significant period of time (25 days on average), while in a small room (8 m² in our unit).

Physical changes, especially during the first hospitalization, are often very significant (alopecia, weight loss, paleness, muscle wasting, skin disorders, etc.). In sterile hematology unit, isolation and, more particularly, the reduction in the number of sensations related to touch, constitute an even more singular context. Everyday life and habits are totally modified (sleeping habits, diet, physical activity, etc.). Physical contact between the patient and their family is very rarely allowed. In this context of hospitalization in a protective environment, the sensations linked to the sense of touch that the patient experiences primarily concern medical treatment, which is invasive for the most part (re-dressing, blood tests, etc.). This daily care is mainly carried out by nurses who are in the front line of the organisation of supportive care initiatives against anxiety.

These circumstances explain the importance for the patient to “stay in touch” with his environment as much as possible [6, 7], and touch-massages® constitute an approach to the patient as a whole, which is not focused exclusively on treating their disease [8–11]. Touch-massages® sessions include a physical and relational dimension that allows the patient to relax and feel reassured, constituting a complementary approach to the medical treatment. Touch-massages® should be adapted to the patient when first taken into care, considering both their overall health, and specific needs at the time of treatment.

Various studies have demonstrated value of massage therapy in improving body representation [12], the vivacity of the sensorimotor system [13]. These different actions could have a positive impact on anxiety and self-representation. Moreover, Diego and Field have shown a positive action on cortisol levels in healthy subjects, marking a decrease in anxiety [14] .

Publications have studied specifically the impact of touch-massage® nursing on different symptoms such as pain or anxiety, in many specialties such as geriatrics [15], cardiology [16], including in intensive care units [17, 18], in oncology [19]^,^ and notably in the context of chemotherapy-related fatigue [20]. But these publications remain rare, particularly in terms of randomized controlled studies, whereas this practice is widespread in all sectors of health care.

We therefore wished to measure the impact of touch-massage® on the anxiety and self-esteem of patients with severe hematological pathologies, hospitalized in sterile hematology unit, in comparison with patients benefiting from a quiet time, thanks to the first randomized controlled study carried out in this specific context.

## Methods

### Study design and participants

We conducted a randomized controlled study with an experimental group (receiving touch-massage® nursing) and a control group (receiving quiet time). The primary endpoint was based on the mean difference in the Anxiety-Status score before and after each session. The impact of the massage sessions on self-esteem was the secondary endpoint.

### Sample

All patients hospitalized in the sterile hematology unit of a University Hospital between January and December 2015 and who met the eligibility criteria for participation in the study were invited to participate in the research.

### Eligibility criteria

#### Inclusion criteria

The inclusion criteria concerned patients with hematological diseases (leukemia, myeloma, lymphoma) hospitalized in a sterile hematology unit for at least 3 weeks, aged 18 to 65 years, female or male, who had never had nurse touch-massage® care.

#### Exclusion criteria

The exclusion criteria concerned patients with an allergy to sweet almond oil, skin lesions on the back and/or arms and/or hands, cognitive and/or psychiatric disorders preventing responses to the interviews and/or questionnaires, and the physical impossibility to remain in a sitting position on an ergonomic chair for the touch-massage® .

### The interventions - massage group and control group

#### Touch-massage® group

Each patient in the massage group received three sessions of touch-massage® over a 3-week period (one session of 15 min per week) by the same nurse trained in touch-massage®, in the calm of the patient’s room.

The massage was performed directly in contact with the skin, using sterile massage oil. The patient was installed on an ergonomic chair to promote the comfort of both the patient and the nurse.

Each massage took place according to a previously established protocol, consisting of a massage of the back, arms and scalp (in Supplementary Material 1). The touch-massage® includes gentle, gliding, and enveloping motions, with alternating rhythms. The three nurses in this study had previously been trained in touch-massage® (175 h of training) [8, 9].

#### Control group

The participants of the control group were invited to have a quiet time sitting on an ergonomic chair in their room without being disturbed, for the same duration and frequency as the patients included in the touch-massage® group; one period of 15 min per week for 3 weeks.

### Randomization

Randomization between the two groups was 1:1, using a software (Capture system ®) without stratification.

### Outcomes

The following data were collected in order to describe the two group samples: sex, age, hematological pathology, and first hospitalization or not in a protected sector. Each patient completed the State-Trait Anxiety Inventory (STAI) validated in French [21, 22] in order to evaluate primarily the predisposition of each subject to anxiety (anxiety-trait subscale STAI-T). It is a self-report questionnaire aimed at studying the anxiety-trait STAI-T (personality trait of the patient, their usual anxiety). It consists of 20 questions, all items are rated on a 4-point scale. The patient assigns a score to each question. The scores range from 20 to 80. The higher the score, the greater the anxiety personality trait. The results can be categorized as follows: very high anxiety (score > 65), high (score between 56 and 65), medium (score between 46 and 55), low (score between 36 and 45), very low (score < or equal to 35).

#### Primary endpoint: changes in anxiety before and after each session

In the treated group, anxiety was assessed one hour before and one hour after each touch-massage® session, using the State-Trait Anxiety Inventory [21], a self-report questionnaire designed to study anxiety-state (anxiety at a given moment) by anxiety-state subscale STAI-State. It is composed of 20 questions, for each item is rated on a 4-point scale. The patient assigns a score to each question. The scores range from 20 to 80. The higher the score, the greater the anxiety. The results can be categorized as follows: very high anxiety > 65, high from 56 to 65, average from 46 to 55, low: from 36 to 45, very low < or = 35.

In the control group, anxiety was assessed in the same way before and after a 15-minute quiet period.

For each patient, the difference in the STAI-State score before and after each massage session (or quiet period) was calculated.

#### Secondary endpoints: self-esteem

Each patient completed the self-esteem questionnaire before the first touch-massage® session and after the last touch-massage® session. The impact of the touch-massage® sessions on self-esteem was assessed by the difference in the overall score on the Rosenberg Self-Esteem Scale [23]. This is a self-report questionnaire composed of 10 items, rated from 1 to 4. The scores vary from 10 to 40. The higher the score, the higher the self-esteem.

### Number of subjects required and statistical methods

As no study on the evaluation of this therapy in the context of this pathology has been published, we relied on internal data for this study. We sought a decrease in the STAI-State score after one massage session of 8.8 points with a standard deviation of 2. We wished to demonstrate a difference between the two groups of 8 points. With alpha and beta risk set at 5% and 20%, 58 patients were needed. In order to guarantee the power of the study, a total of 62 patients were recruited.

All analyses were performed on the intention-to-treat population. They were completed by a per protocol analysis.

The difference in score at each session was modeled using a linear mixed model taking into account the group effect (“touch-massage® " vs. “no massage”), time (i.e. session), and time/group interaction. A random patient effect was also taken into account given the repetitive nature of the data. The Rosenberg Self-Esteem Questionnaire score was compared using a linear model based on the score measured at baseline.

Analyses were performed using R software version 3.5.1.

### Ethic

The trial was performed in accordance with the Declaration of Helsinki 1996. All patients provided written informed consent. The study was approved by the Comité de Protection des Personness OUEST IV Nantes (decision n°518/2014). The trial was registered : Trial registration NCT02343965 (22/01/2015).

## Results

### Patient characteristics and study flow diagram

**Fig. 1:**
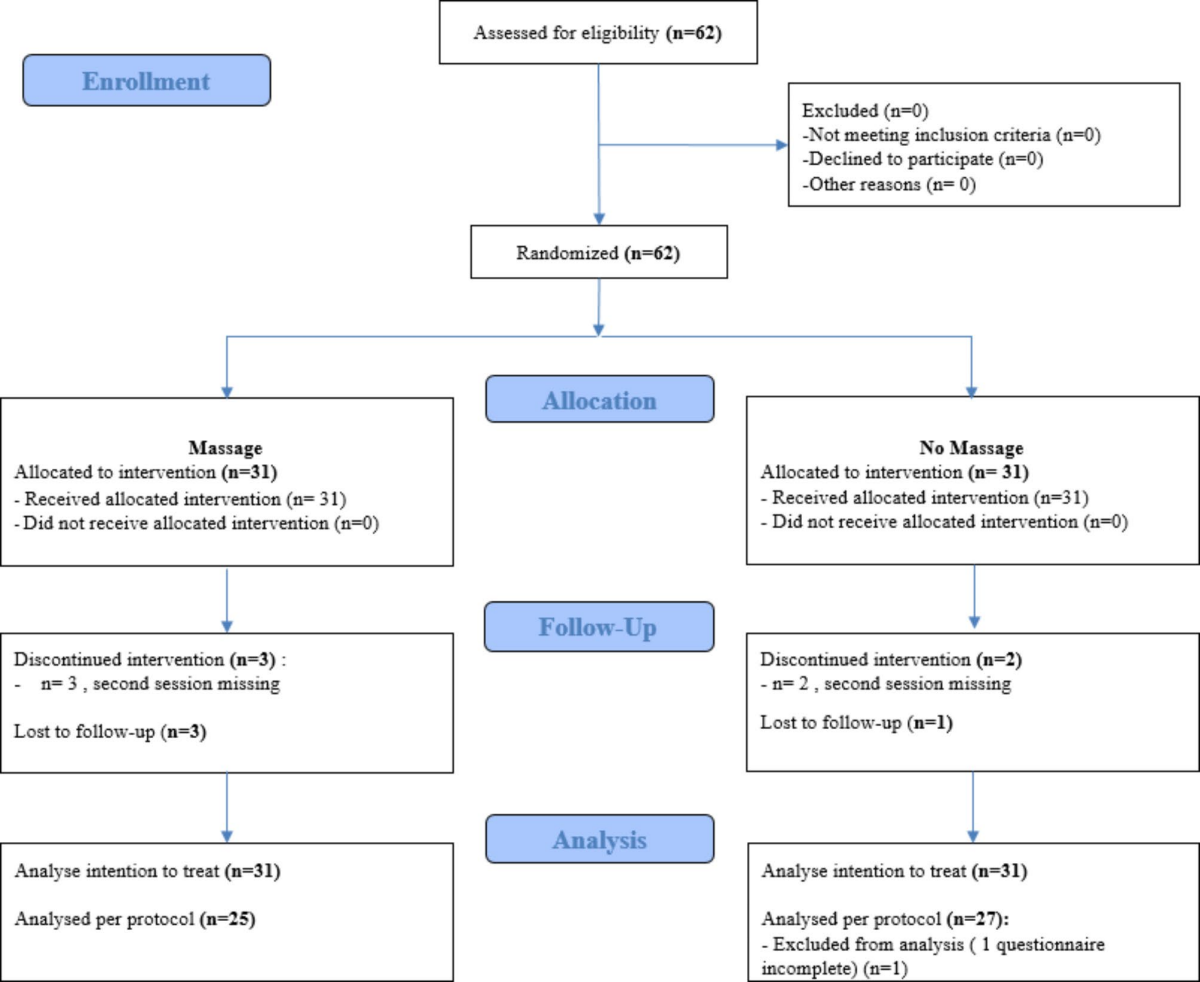
Study flow diagram

All eligible patients were recruited (Fig. 1), once randomized, they all had the three sessions of either massage or quiet time. We had 3 premature discharges (after S2) in the massage group and 1 premature discharge (after S1) for the no massage group due to discharge from the protected sector. Data losses were as follows: in the massage group, 3 patients did not attend session 2, and in the massage group 2 patients did not attend session 2, and one questionnaire was incomplete (S3).

The majority of patients were men with leukemia, a little more than half of whom had already been hospitalized in a protected sector. The average anxiety score was 43.4, corresponding to patients with an anxious personality considered to be of low intensity (Table 1). No significant inconveniences or undesired effects in each group were reported.

**Table 1:** Patient Characteristics

	No massage (N = 31)	Massage (N = 31)	Total (N = 62)
**Age, mean (SD), years**	49.7 (11.8)	51.3 (13.2)	50.5 (12.5)
**Female n (%)**	9 (29.0%)	15 (48.4%)	24 (38.7%)
**Pathology**			
**Leukemia**	27 (87.1%)	26 (83.9%)	53 (85.5%)
**Myeloma**	1 (3.2%)	0 (0.0%)	1 (1.6%)
**Lymphoma**	3 (9.7%)	5 (16.1%)	8 (12.9%)
**First hospitalisation**	16 (51.6%)	18 (58.1%)	34 (54.8%)
**State Trait Anxiety**	43.5 (8.7)	43.3 (10.1)	43.4 (9.4)

### Efficacy in relation to primary outcome: change in anxiety status

**Table 2:** Mean difference in anxiety-status score in intention-to-treat and per protocol

	No massage	Massage	Difference	*p* value
STAI State (Intention to treat)	-0.92[-3–1.16]	-11.52[-13.6 - -9.43]	10.6[7.65–13.54]	< 0.001
STAI State (Per protocol)	-0.59[-2.79–1.6]	-11.51[-13.79 - -9.22]	10.91[7.75–14.08]	< 0.001

The improvement in patient state anxiety score was statistically significant in the treatment group compared to the control group: a mean score decrease of 10.6 [7.6–13.5] on the Spielberger State Trait Anxiety Inventory (STAI-State) was obtained for the massage group (p ≤ 0.001) (Table 2).

**Fig. 2:**
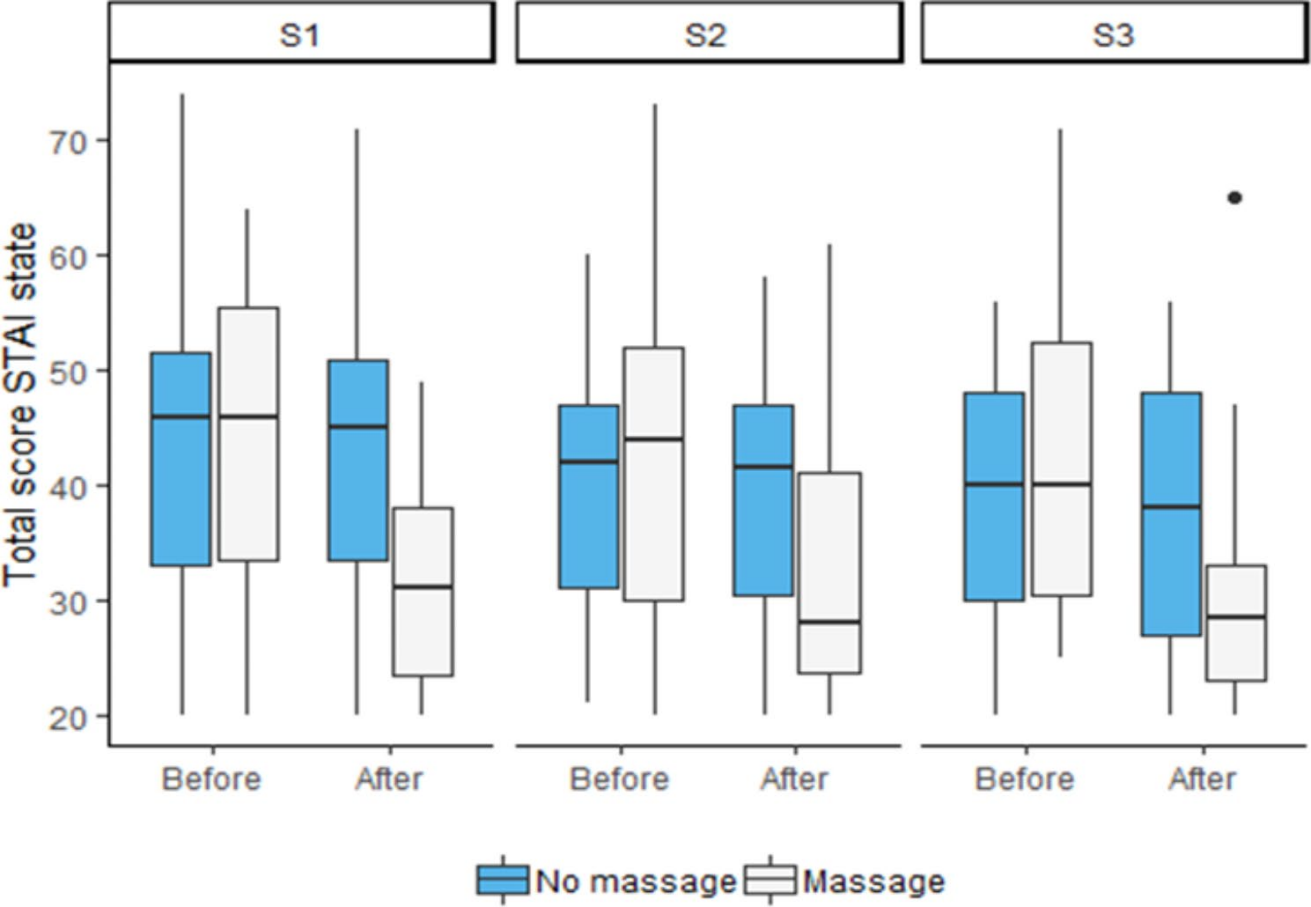
Changes in anxiety scores before and after each of the three sessions (S1,S2,S3)

There was a clear decrease in anxiety after each touch-massage® session (Fig. 2; Table 3), with a change in categorization of anxiety level for the massage group in contrast to the control group.

**Table 3:** Changes in anxiety scores before and after each of the three sessions (S1,S2,S3)

		Score STAI state		
		No Touch-massage®	With Touch-massage®	Total
		N = 31	N = 31	N = 62
Session 1 before	N	31	31	62
	Median [Q1-Q3]	46.0[33.0;52.0]	46.0[33.0;56.0]	46.0[33.0;53.0]
Session 1 after	N	31	31	62
	Median [Q1-Q3]	45.0[33.0;51.0]	31.0[23.0;38.0]	36.0[27.0;46.0]
Session 2 before	N	28	29	57
	Median [Q1-Q3]	42.0[31.0;47.0]	44.0[30.0;52.0]	43.0[31.0;50.0]
Session 2 after	N	28	28	56
	Median [Q1-Q3]	41.5[30.0;47.0]	28.0[23.5;41.0]	36.5[24.5;44.5]
Session 3 before	N	29	27	56
	Median [Q1-Q3]	40.0[30.0;48.0]	40.0[30.0;53.0]	40.0[30.0;49.0]
Session 3 after	N	29	28	57
	Median [Q1-Q3]	38.0[27.0;48.0]	28.5[23.0;33.0]	30.0[25.0;42.0]

(NB: the number of participants is variable due to patients lost to follow-up, unrealized sessions and an incomplete questionnaire)

### Secondary endpoints: self-esteem

**Table 4:** Assessment of self-esteem by patients

	No massage	Massage	Difference	*p* value
Self-esteem	24.42[23.45–25.39]	25.01[24.04–25.98]	-0.59[-1.96–0.79]	0.4

The improvement in self-esteem score was not significant (Table 4) in the treated group compared with the control group, although patients reported in interviews the importance of massage in maintaining self-esteem.

## Discussion

The results of our study show that the touch-massage® intervention by a trained nurse significantly reduces the intensity of anxiety in patients hospitalized in a protected hematology unit. We were unable to demonstrate an impact on self-esteem.

### Comparison with the literature

In bone marrow transplantation [24] and oncology [25], it is important to know more about supportive care, which has been proven to improve the difficult experience of these hospitalizations and to improve patient symptoms.

The results of our study are in line with those carried out in the general population where this therapy has also helped to improve patient anxiety and stress [14, 26, 27] ,allowing for a reduction in the use of healthcare services [18, 28].

Our study confirms and reinforces the results of different studies of lesser quality carried out specifically in these hospitalization sectors and in this very specific population. A small study showed a decrease in cortisol and prolactin (known to increase under stressful conditions) [26] but these results are debated [29]. A controlled feasibility study on 20 patients [30] and an uncontrolled pilot study [31] showed a decrease in various symptoms including anxiety. Two other studies have been carried out in adults [32] and in pediatrics [33] without significant results (but with small samples) despite qualitative interview data underlining the positive experience of this care [34]. More recently, a retrospective study published in 2023 highlighted that this therapy seems to reduce acute pain, stress and anxiety, among patients in acute hematological and/or oncological situations [35].

As part of a more comprehensive approach, this therapy could also be offered to a patient’s relatives. This aspect was considered in a study in 2002 [36], and the results showed a significant decrease in anxiety scores, depression and general fatigue concerning the massage group.

Moreover, it may be beneficial to study the effect of massage on caregivers in these hospital units who are themselves subject to intense stress [37, 38].

In the same vein, the massage training of patients’ relatives could be envisaged, as suggested by the results of a study carried out on the caregivers of American veterans [39].

Apart from the impact on anxiety, this therapy could have other benefits for the patient such as cognitive and self-image improvements [12, 13] even if we were unable to demonstrate an impact on self-esteem. Pilot studies have shown in other populations an action on the immune system, which could be of interest in the context of bone marrow transplantation [40–42]. Other studies have highlighted effects on other physical or psychological symptoms such as pain, mood and fatigue in patients with metastatic or non-metastatic cancer, in both survivors and those in palliative care [43–45].

Finally, it could also be beneficial to evaluate if the effect of massage on anxiety persists over time and if it enables the occurrence of other symptoms during hospitalization and afterwards to be reduced (or even be prevented). For example, El-Jawahri [46], has shown the value of integrating palliative care with transplant care, which improves the symptoms of depression and post-traumatic stress disorder six months after transplantation. The reduction of the burden of symptoms and anxiety during transplantation partly explains the effect of this type of intervention.

### Limitations of the study

This is a single-center, unblinded study. Blindness being a recurrent problem in massage therapies, the use of a questionnaire allows to standardize the evaluation measure. Another limitation of this study is the choice of the right dose-response. few studies have investigated the right dosage of massage to obtain effects. A recent study has, however, looked at these effects on a population with similar objectives [47]. Future studies should clarify this question.

It was not possible to evaluate the drug and non-drug treatments concomitant with the protocol. Treatments with anxiolytic objectives could have had an impact on the results, even if randomization made it possible to reduce this risk.

Furthermore, although the decrease in anxiety of the hospitalized patients was significant in the massage group, the impact on self-esteem was not significant, possibly because the number of patients was too small to show a significant result on this criterion.

### Strengths of the study

To our knowledge, this is the first and only randomized controlled study evaluating the value of touch-massage® in the protected haematology sector, which shows a significant impact of this non-medicinal intervention on the reduction of anxiety in patients. Its implementation is simple, carried out by a major player in these services, but requires a change of point of view on physical contact to be developed in the protected hematology sector, where contact is reduced to a strict minimum [7]. In this pragmatic study, three nurses were trained to perform the touch-massage®, which was standardized in order to avoid an effect related to each nurse’s own practice. Moreover, we were able to enroll the necessary number of subjects, which is often complicated in these fragile populations and in this type of study [48].

## Conclusion

This research provides evidence for caregivers to integrate touch-massage® into the nursing procedures to be proposed to patients in the context of isolation experienced in sterile hematology unit. We know that the reduction of psychological symptoms during this type of hospitalization can reduce long-term psychological complications [46].

The organization and training of nurses in complementary therapies such as touch-massage® remains to be established in sectors with numerous technical medical procedures.

The care for the relatives of these patients, and even the implementation of massages by the caregivers, are also other avenues for reflection.

### Electronic supplementary material

Below is the link to the electronic supplementary material.

Supplementary Material 1

## Data Availability

The datasets used and/or analysed during the current study are available from the corresponding author on reasonable request.
