# Supplementary material for: Impact of the practice of touch-massage® by a nurse on the anxiety of patients with hematological disorders hospitalized in a sterile environment, a randomized, controlled study
Source: BMC Complement Med Ther. 2024 Jan 2;24:1. doi: 10.1186/s12906-023-04302-3 (PMC10759707; doi:10.1186/s12906-023-04302-3)
Supplement: Supplementary file 1 — Supplementary Material 1 [file 12906_2023_4302_MOESM1_ESM.docx]

**Supplementary Material 1:** Protocol for the Touch-Massage® session

**STEP 1 : Installation of the patient (1 min. 30 seconds)**

A disposable protective tissue is attached to the headrest of the chair, and sterile towels are placed on the chest and arm supports.

To help with the installation, the patient is invited to remove the top of their pyjamas when they are seated in the chair: after having ensured the safety of the patient, the nurse places herself behind them, in order to preserve the patient's intimacy as much as possible, in particular concerning the respect of women's modesty with regard to their breasts. This positioning allows the caregiver to avoid being face to face with the patient at any time in order to preserve the patient's privacy.
The nurse positions a sterile towel on the lower back of the patient, just above the waist of the pyjama bottoms.

**STEP 2: Contact (30 seconds)**

The nurse stands behind the patient.

The nurse's hands are first positioned on the patient's shoulders, then the patient's back, arms, and hands are lightly oiled with sweet almond oil.

**STEP 3: Toch-Massage® of the back (10 min.)**

1- Fluid movements, gentle sliding pressure using the full palm of the hand.

˃ A back and forth motion along the back alternating one hand then the other

˃ A back and forth motion along the back with both hands simultaneously

These maneuvers are repeated 3 times with a slow and regular rhythm

2- Positioning of the nurse on one side of the patient

˃ Light kneading of the trapezium

˃ The patient's arm is released from the chair and held by the nurse for a gentle mobilization

˃ A few light sliding pressures are applied to the whole arm and hand

˃ Opening of the palm of the hand, slight stretching of each finger

˃ The arm is repositioned on the chair

3- The nurse places herself on the other side of the patient

4- Massage of the trapezium, the arm and the hand following the same procedure.

5- Positioning of the nurse in front of the patient

6- Gentle alternating pressure on the patient's shoulders

7- Light kneading of the shoulders

8- Positioning of the nurse behind the patient

Connecting gestures are practiced regularly throughout the touch-massage®.

**STEP 4: Final maneuvres (3 min.)**

1- Gentle massage on the whole body

˃ Of the back, arms, and hands

˃ The nurse covers the patient's back with the sterile towel placed at the bottom of the patient's back at the beginning of the treatment, this provides a pleasant sensation for the patient and also removes the oil remaining on the surface of the skin

2- Information at the end of the Touch-massage® session

˃ The nurse helps the patient to get up from the ergonomic chair in complete safety after having helped them to put on their pyjama jacket, using the same precautions as when the patient takes off their pyjama jacket

˃ The nurse takes leave of the patient


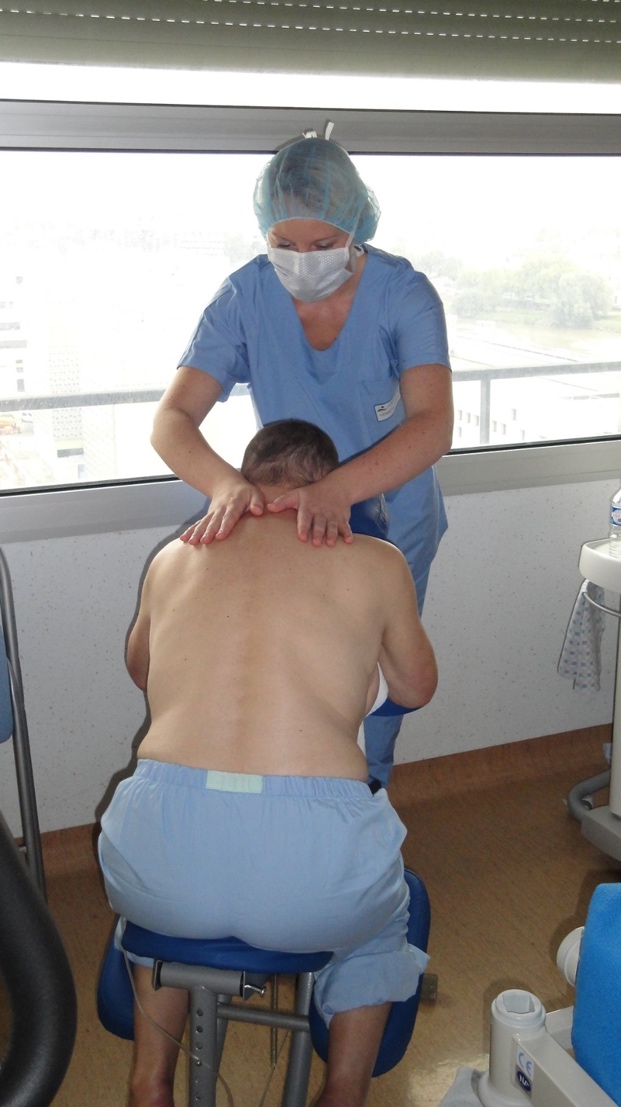


**Figure S1: Step 3: Back Touch Massage** *(Position of the nurse in front of the patient*

*Gentle full palm pressure on the patient's shoulders)*
